# Social disadvantage during pregnancy: effects on gestational age and birthweight

**DOI:** 10.1038/s41372-023-01643-2

**Published:** 2023-03-13

**Authors:** Joan L. Luby, Sarah K. England, Deanna M. Barch, Barbara B. Warner, Cynthia Rogers, Christopher D. Smyser, Regina Triplett, Jyoti Arora, Tara A. Smyser, George M. Slavich, Peinan Zhao, Molly Stout, Erik Herzog, J. Philip Miller

**Affiliations:** 1 Department of Psychiatry, Washington University School of Medicine, St. Louis, MO USA; 2 Department of Obstetrics and Gynecology, Center for Reproductive Health Sciences, Washington University School of Medicine, St. Louis, MO USA; 3 Department of Psychological & Brain Sciences, Washington University in St. Louis, St. Louis, MO USA; 4 Department of Pediatrics, Washington University School of Medicine, St. Louis, MO USA; 5 Department of Neurology, Washington University School of Medicine, St. Louis, MO USA; 6 Division of Biostatistics, Washington University School of Medicine, St. Louis, MO USA; 7 Department of Psychiatry and Biobehavioral Sciences, University of California, Los Angeles, CA USA; 8 Department of Obstetrics and Gynecology, University of Michigan, Ann Arbor, MI USA; 9 Department of Biology, Washington University in St. Louis, St. Louis, MO USA

**Keywords:** Paediatrics, Health care

## Abstract

**Objective:**

Whether psychosocial adversity during pregnancy impacts fetal health outcomes at birth remains underexplored. This is a critical issue given significant social disadvantage and psychosocial stress faced by pregnant women worldwide.

**Study design:**

Measures of social disadvantage and psychological factors, and medical/reproductive and nutritional health status in pregnant women were obtained at each trimester. Using Structural Equation Modeling (SEM), we investigated the relationship of forms of adversity to each other and to infant gestational age, and birthweight.

**Results:**

Among 399 singletons, Social Disadvantage significantly predicted gestational age (*p* = 0.003), and residual birthweight (*p* = 0.006). There was a 0.4 week decrease in gestational age and a 3% decrease in birthweight for each standard deviation increase in Social Disadvantage.

**Conclusion:**

Significant negative effects of social adversity on the developing fetus were found. Notably, these effects emerged despite good prenatal care and after accounting for maternal age and medical reproductive risk factors.

## Introduction

The theory of the developmental origins of health and disease (DOHaD) has focused scientific attention on the powerful impact of the intrauterine environment on neonatal health outcomes [1]. Beyond the well-established effects of maternal physical health and nutritional status, as well as drug and toxin exposure, more recent literature has emphasized the material importance of the maternal psychosocial environment on infant outcome, focusing on experiences of adversity and stress [2]. Numerous studies have documented significant effects and explored the mechanisms by which such psychosocial factors, conceptualized as “prenatal programming”, relate to infant outcomes. These studies emphasize the role of maternal psychological well-being, and have focused on the effects of stress and depression on the developing fetus.

A complex issue central to investigations of the effects of psychosocial adversity on health outcomes is the frequent co-occurrence of numerous forms of adversity, making it difficult to determine whether there are specific effects of particular types of exposure on developmental outcomes. Substantial research has examined the impact of maternal depression and related psychological stress during pregnancy on infant outcomes [3], but this work has not yet attempted to dissociate these factors from poverty or other forms of social stress [4]. Therefore, it remains unclear if there are distinct effects of social disadvantage on prenatal programming that can be differentiated from the established effects of maternal stress and/or depression during pregnancy. Notably, outside of maternal health, diet, and mental health and stress, few studies have focused on the effects of poverty and related forms of prenatal social disadvantage on key infant outcomes such as gestational age and birthweight. Many studies have established that women living in poverty face higher rates of preterm birth [5]. However, which elements of disadvantage exposure (e.g., diet, health, lack of prenatal care, or psychosocial stress) are most strongly related to these negative fetal outcomes remains unclear.

In an ongoing longitudinal study, we collected data from pregnant women in each trimester of pregnancy to determine how a variety of forms of social disadvantage and psychological factors such as depression, psychosocial stress, and stressful and traumatic life events (combined across pregnancy and by trimester) affect neonatal outcomes after accounting for key health parameters. Here, we focus on gestational age and birthweight (accounting for gestational age) as key neonatal outcomes known to be associated with later child health and development [6, 7]. We also examined other perinatal outcome variables of interest including NICU stay and breastfeeding metrics. Gestational age is a robust predictor of later health and developmental outcomes, not only in the context of early preterm birth but also in the cases of late moderate preterm and early term birth [8]. Birthweight is also one of the first indicators of later health and developmental outcomes with extremes of birthweight, such as small for gestational age (SGA; <10th percentile at birth) and large for gestational age (LGA; >90th percentile at birth) status, established as sensitive markers of cardiometabolic and neurodevelopmental risk into adulthood [9, 10]. This study aims to fill this gap by examining the relations of maternal medical, psychosocial (perceived stress, discrimination), and psychological factors, (i.e., anxiety, depression), and social determinants of health (i.e., income to needs, nutrition, neighborhood) to gestational age and birthweight, after accounting for key aspects of maternal reproductive health (i.e., maternal medical risk factors, cervical length).

## Methods

The current study, Early Life Adversity Biological Embedding and Risk for Developmental Precursors of Mental Disorders (eLABE), is a multi-wave, multi-method NIMH-funded study designed to investigate mechanisms by which prenatal and early life adversity impact infant neurodevelopment. Pregnant women who were participants in a study of preterm birth within the Prematurity Research Center at Washington University in St. Louis with no alcohol or substance use during pregnancy (except tobacco or marijuana) and without known pregnancy complications or fetal congenital problems were invited for eLABE participation. The study recruited 395 women during pregnancy (*N* = 268 eligible subjects declined) and their 399 singleton offspring (4 mothers had 2 singleton births during recruitment). Out of those originally invited and interested, 26 were ineligible (13 screened out prior to consent and 13 consented subjects were ineligible due to later discovery of substance abuse or a congenital anomaly). Women facing social disadvantage were over-sampled by increased recruitment from clinics serving low-income women leading to a sample enriched for preterm infants (<37 weeks’ gestation, *N* = 51 born preterm). Of the 399 pregnancies, 50 reported tobacco use during pregnancy, and 49 reported cannabis use (20 reported both). See *Supplemental Materials* (Tables S4 and S5) for analyses that controlled for use of cannabis and tobacco.

Maternal depression, experiences of stress, as well as demographic information including insurance, education, address, and household composition were obtained from participants at each trimester during pregnancy. Mothers were followed prenatally as a part of the March of Dimes Study of Prematurity (MOD) study and all mothers engaged in prenatal visits [7]. Standard transvaginal ultrasound images of the cervix for length (known risk factor for preterm birth) were obtained and the 3^rd^ trimester measure was used in the analyses [7]. Mothers and their newborns were invited for an assessment shortly after birth which included neonatal MRI during which mothers completed a comprehensive measure of life stress, trauma and discrimination. Measures are detailed in Table S1. All study procedures were pre-approved by the Washington University School of Medicine Institutional Review Board and written consent was obtained from all study mothers.

With the aim of investigating and disentangling the high levels of correlation between various forms of adversity, Structural Equation Modeling (SEM) was used to test the differential relationships of types of adversity to gestational age and birthweight (adjusted for gestational age). We used a range of variables to define two latent factors, Maternal Social Disadvantage and Maternal Psychosocial Stress, as well as measures of other important potential sources of variance, such as maternal health parameters including medical risk, body mass index, maternal age, and short cervix. We then examined whether we could identify differential relationships of these two factors to either gestational age or birthweight (as well as other outcomes), all targeted because of their importance to long-term health and development.

The following datapoints were used to estimate Social Disadvantage (see *Supplemental Materials* for details): household Income to Needs Ratio (I/N) in each trimester, national Area Deprivation Index (ADI) percentiles [11], Healthy Eating Index (HEI) [12], highest level of educational attainment, and health insurance status. To estimate Psychosocial Stress, four measures were used (see *Supplemental Materials* for details): Perceived Stress Scale (PSS) in each trimester [13], Edinburgh Postnatal Depression Scale (EPDS) in each trimester [14], Stress and Adversity Inventory (STRAIN, lifetime count and severity which also accounts for social support and marital status) at time of neonatal scan (*n* = 190) or at follow-up at one or two years (*n* = 80) [15], and Everyday Discrimination Scale (EDS) at time of neonatal scan [16]. Within this cohort, race, a socially-defined construct, was highly correlated to Social Disadvantage Factor indices, offering no additional improvement to the model after other variables (including racial discrimination) were accounted for; therefore race was not included as a variable.

### Maternal medical factors

To control for maternal medical risks that might be confounded with social or psychological disadvantage, maternal age at delivery (Table 1) and pre-pregnancy body mass index (Table 1) from first prenatal visit was added to the model. In addition, a Maternal Medical Risk Score (MMR), containing pre-existing and pregnancy related medical conditions, was computed using a validated measure of maternal medical comorbidities from the medical record accounting for 22 medical conditions weighted by severity (an index of > 3 associated with severe morbidity) [17, 18]. Because of the potential for a direct effect of maternal nutrition on birthweight, a direct link was included for HEI. Participants had on average five prenatal visits. Nine neonates had mothers with a cervical length <2.5 cm, deemed “short cervix”. [19]

**Table 1:** Means and Standard Ds of Variables included in SEM Model.

Variable	*n*	Mean (SD) or *N* (%)	Min	Max
*Disadvantage Indices*				
Log_10_ (Income/Needs—I/N)^a^				
1st Trimester	385	0.24 (0.40)	−0.495 (0.32)^d^	1.096 (12.5)^d^
2nd Trimester	305	0.28 (0.41)	−0.523 (0.30)	1.096 (12.5)
3rd Trimester	330	0.26 (0.41)	−0.456 (0.35)	1.096 (12.5)
ADI—Area Deprivation Index—(National Percentile)	376	69.09 (24.84)	1	100
HEI (Healthy Eating Index)–2016 Total Score	308	58.45 (9.90)	31.7	80.7
Health Insurance^c^	399		–	–
Individual/Group		200 (50%)		
Medicaid		145 (36%)		
Medicare		7 (2%)		
Uninsured		45 (11%)		
VA/Military		2 (1%)		
Education	355			
Less than high school		28 (7%)		
High school grad		196 (55%)		
College grad		56 (16%)		
Post-grad degree		77 (22%)		
*Psychosocial Stress Indices*				
Edinburgh Postpartum Depression Scale (EPDS)				
1st Trimester	396	5.25 (4.88)	0	25
2nd Trimester	331	5.00 (4.94)	0	26
3rd Trimester	330	4.38 (4.70)	0	25
Perceived Stress Survey (PSS)				
1st Trimester	394	13.69 (7.39)	0	38
2nd Trimester	304	13.81 (7.68)	0	38
3rd Trimester	325	13.25 (7.36)	0	37
Discrimination Survey^b^	366	1.490 (0.88)	1	6
STRAIN (stressful life events)				
STRAIN-CT (count)	363	7.62 (6.06)	0	30
STRAIN-WTSEV (weighted severity)	363	22.67 (19.87)	0	99
*Maternal Medical Factors*				
Maternal Medical Risk Score	399	1.28 (1.69)	0	12
Pre-Pregnancy BMI	307	29.05 (8.34)	16.1	64.6
Maternal age at delivery	399	29.23 (5.32)	18.65	41.90
Cervical Length	358	40.40 (7.61)	4.5	69.2
*Birthweight Relevant Variables*				
Birthweight	399	3129 (609)	860	4665
Log_10_(Birthweight)	399	3.49 (0.10)	2.93	3.67
Child Sex	399		–	–
Male		221 (55%)		
Female		178 (45%)		
Gestational Age	399	38.31 (1.99)	26.4	41.6
Log_10_(Birthweight) residual	399	0.00 (0.06)	−0.22	0.20

^a^Log transformed because of skewed distribution of variable and to make the ratio symmetric around 1.0.

^b^Score only if perceived as racial in nature, 0 otherwise.

^c^Analyzed as Individual/Group vs. all others.

^d^Untransformed values.

### Child variables

We used gestational age as determined by best obstetric estimate using last menstrual period or earliest ultrasound dating available at birth. In addition, birthweight was collected from the medical record at delivery. For secondary analyses, Neonatal Intensive Care Unit (NICU) admission and NICU breast milk use was extracted from the electronic medical record. For additional secondary analyses, breast milk feeding data was obtained on all infants by parental report based on the Center for Disease Control Infant Feeding Practices II study food frequency checklist [20] at four months and 1 years of age.

### Statistical methods

**Fig. 1:**
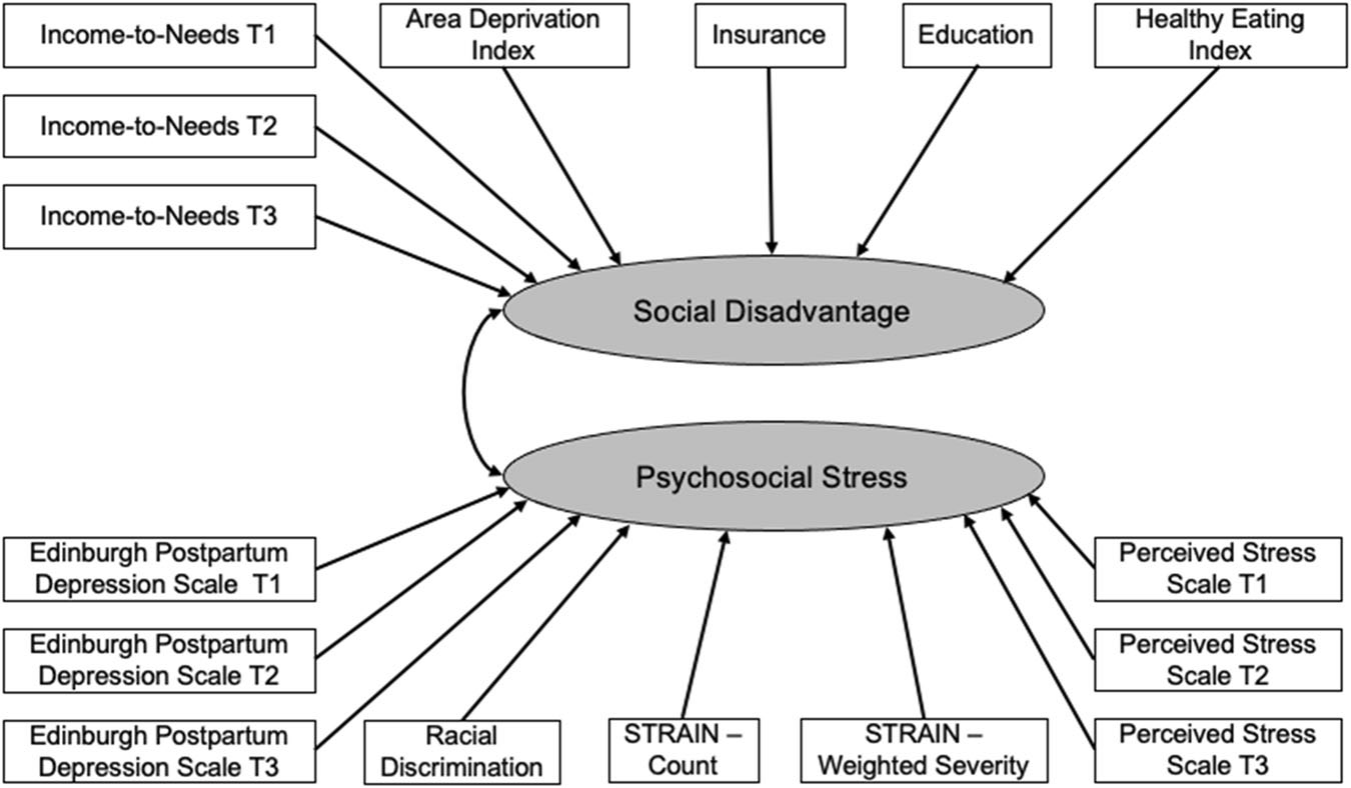
Structural Equation Model. This figure illustrates the conceptual structural equation model. Please see Table S2 for factor loadings from the computed structural equation model. The factor loadings for the model predicting gestational age are used for illustration, but as can be seen in Tables S2,S3, the loadings for the model predicting birthweight controlling for gestational age are almost identical.

We hypothesized that we could dissociate, and find significant independent relationships of, Maternal Social Disadvantage versus Maternal Psychosocial Stress to gestational age and birthweight as well as secondary outcomes. To test this hypothesis, we conducted confirmatory factor analyses for a priori hypotheses (depicted in Fig. 1, additional details in *Supplemental Methods*) about which indicators loaded on these two factors using the MPlus software to validate our grouping of prenatal adversity variables into a Social Disadvantage factor (I/N, ADI, Insurance Status, Maternal Education, and Maternal Nutrition) and a Psychosocial Stress factor (depression, perceived stress, discrimination, and lifetime measures of trauma and life events) as indicated in Table 1. Education and Insurance were specified as categorical variables within the Social Disadvantage factor.

Once validated, multiple regression SEM models investigated the independent contribution of each latent factor and Maternal Medical Factors in predicting gestational age and birthweight (adjusted for gestational age) in separate models. The Maternal Medical Factors selected to include were the MMRS, maternal age, and pre-pregnancy BMI for both outcomes, cervical length for gestational age and HEI for birth weight. The estimation of the models was with the maximum likelihood method with robust standard errors which is considered robust for non-normality and which allows for estimation in the setting of missing data. Because of reports of differing imprinting by the sex of the fetus [21, 22], we also tested interactions of sex with each latent factor. All computations were done with MPlus version 8.4 or SAS 9.4. In secondary analyses, we used the social disadvantage and psychosocial distress factor scores in logistics regressions with covariates to determine if either predicted NICU stay (controlling for gestational age and birthweight) or breast milk feeding in the NICU (among those children in the NICU) or breast milk feeding at 4 months or 1 year, all coded as binary outcome variables.

## Results

Table 1 presents means and standard deviations for each measure including the Social Disadvantage and Psychosocial Stress factors, as well as maternal medical risk and child outcomes. Although the cohort is weighted toward socially disadvantaged mothers, a broad range across social and psychological variables existed, with 38% of the population having a college degree or higher, over 50% of the population with private insurance, and I/N values extending up to 12 times above the poverty level.

The results of the confirmatory factor analysis are illustrated in Fig. 1 and produced fit indices indicating a good level of fit for the model with two latent factors of Social Disadvantage and Psychosocial Stress; (RMSEA = 0.043, SRMR = 0.055, CFI/TLI = 0.954/0.944). See *Supplemental Methods* for Comparison to other 1 and 3 factor models, which were worse fits. There did not appear to be differential relationships of indicators of either the Social Disadvantage or Psychosocial Stress factors as a function of trimester, with loadings approximately equal across trimesters (Tables S2 and S3).

**Fig. 2:**
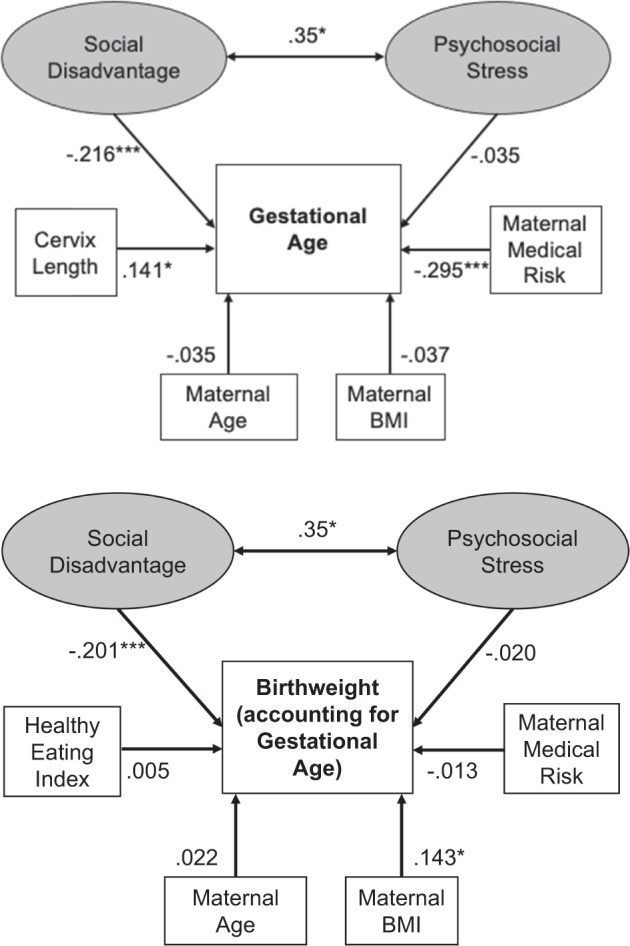
Figure illustrating the relationships of Social Disadvantage and Psychosocial Stress to Gestational Age and Birthweight. Values are standardized estimates paths from the structural equation model. *p < 0.05; ***p < 0.001.

The full SEM model fits are shown in Tables S2-S3. The raw and standardized estimated effects for each model using the latent factors to predict gestational age, birthweight, X, and Y are shown in Table 2 and the significant relationships are illustrated in Fig. 2. The Social Disadvantage latent factor significantly predicted gestational age (*p* = 0.003) and residual birthweight accounting for gestational age (*p* = 0.006). The raw coefficient for gestational age (−0.429) corresponds to approximately a 0.4 week decrease in gestational age for each one standard deviation increase in the Social Disadvantage factor. Similarly, the raw coefficient for birthweight (−0.012) corresponds to a 2.57% decrease in residual gestational age-adjusted birthweight for each standard deviation increase in the Social Disadvantage. In contrast, the Psychosocial Stress factor did not significantly directly predict either gestational age or birthweight, but did have a significant total direct plus indirect effect (*p* = 0.014 for birthweight and *p* = 0.011 for gestational age). The indirect path is primarily through Social Disadvantage. Gestational age was also significantly predicted by MMR and Cervix Length (Table 2), and birthweight was also predicted by pre-pregnancy BMI (Table 2). Social Disadvantage continued to show significant direct effects in predicting gestational age and birthweight in SEM models that accounted for cannabis and tobacco use, both of which were correlated with social disadvantage (See *Supplemental Materials* and Tables S4 and S5).

**Table 2:** Results of Prediction Models for Gestational Age and Birthweight.

	Raw Estimate	Standardized Estimate	Change in Outcome per 1 Standard Deviation Change in Predictor	Raw Estimate	Standardized Estimate	Change in Outcome per 1 Standard Deviation Change in Predictor
Predictors	Gestational Age (GA) Prediction Model			Birthweight Prediction Model (Adjusted for GA)		
Social Disadvantage	−0.429***	−0.216***	0.4 week decrease	−0.012***	−0.201***	2.57% decrease in birthweight
Psychosocial Distress	−0.070	−0.035	NA	−0.001	−0.020	NA
Maternal Medical Risk	−0.348	−0.295	NA	0.000	−0.013	NA
Maternal Age at Delivery	−0.013	−0.035	NA	0.000	0.022	NA
Pre-Pregnancy BMI	−0.009	−0.037	NA	0.001*	0.143*	1.94% increase in birthweight
Cervix Length	0.037*	0.141*	0.3 week increase	NA		
HEI-2016 Total Score	NA			0.000	0.005	NA

*p < 0.05; **p < 0.01; ***p < 0.005.

*HEI* Healthy Eating Index, *BMI* Body Mass Index.

We next tested interactions of sex with each latent factor. There were no significant interactions of sex with Social Disadvantage for either gestational age (*p* = 0.922) or birthweight (*p* = 0.813). Similarly, there were no significant interactions of sex with Psychosocial Stress for either gestational age (*p* = 0.490) or birthweight (*p* = 0.416).

Given the significant financial costs related to NICU care, in secondary analyses, we used the estimated factor scores for Social Disadvantage and Psychological Stress and examined their relationship to the need for NICU admission using logistic regressions including both factors and relevant covariates. As shown in Table S6, neither Social Disadvantage or Psychosocial Stress predicted NICU stay after controlling for gestational age and birthweight. We also examined relationships of each factor score to breast milk feeding practices among infants who were in the NICU. For infants admitted to the NICU, neither Social Disadvantage or Psychosocial Stress predicted the use of any breast milk feeding (ever). However, Social Disadvantage, but not Psychological Stress, predicted breastmilk feeding at NICU discharge, as well as breastmilk feeding at 4 months and 1 year among infants in the sample as a whole (Table S6).

## Discussion

The findings from these analyses demonstrate the central relationship of Social Disadvantage, a latent factor that includes income to needs, insurance status, education, area deprivation, and maternal nutrition, to both gestational age and birthweight even after accounting for maternal age and medical reproductive risk factors. In secondary analyses, we explored whether these factors were associated with NICU stay and breast-feeding practices. Although previous studies have focused on how prenatal economic or psychological stressors contribute specifically to gestational age and low birthweight outcomes [23, 24], we are unaware of others that have examined their contribution when combined and accounting for markers of reproductive risk. Our examination of both latent factors simultaneously allowed us to distinguish the importance of Social Disadvantage factor as distinct from Psychosocial Stress in contributing to gestational age and birthweight. Effects of the SD factor on both gestational age and birthweight as well as breast feeding at NICU discharge (among NICU babies) and throughout the first year of life (within the whole sample) were found. No independent effects on need for NICU stay or breast feeding in the NICU were found when controlling for gestational age and birthweight.

The clinical significance of this is that for dyads in the lowest quartile of estimated Social Disadvantage factor, the average gestational age was 39.0 weeks, while the highest quartile had a mean gestational age of 37.7 weeks, a difference of almost 9 days, a very significant duration in fetal development in predicting risk for later health and developmental trajectories [6]. While the importance of gestational age has been well established in infant outcomes, birthweight is also a critical early indicator of risk for later cardiometabolic and neurodevelopmental risk into adulthood [9, 10], as well as child and adult cognitive and educational outcomes [6, 25].

This Social Disadvantage factor predicted these key neonatal metrics even when other critical markers of health and known predictors of birthweight are accounted for in the models. Disadvantage is a robust risk factor for both shorter gestation and lower birthweight relative to gestational age, suggesting it should become a central clinical prevention target in pregnancy. The only other significant direct predictors of gestational age were MMR and cervical length, and the only other direct predictor of birthweight was pre-pregnancy BMI. This combination of results pertaining to gestational age and birthweight elucidates the critical role that Social Disadvantage experienced during pregnancy plays in fetal outcomes. They elevate the importance of Social Disadvantage and underscore the need for more focused public health attention to social disadvantage in pregnancy given the clear impact on neonatal birth outcomes, with lower gestational age and birthweight a harbinger of poor physical, cognitive, and emotional health later in development [8].

These results continue to be amplified postnatally. Social Disadvantage diminishes breast milk feeding in and out of the NICU, both factors that exacerbate long term health outcomes often seen in economically disadvantaged populations [26]. Within the NICU, psychosocial adversity does not impact breast feeding initiation within our cohort. However, over the NICU stay, Social Disadvantage becomes negatively related to breast milk use, a finding similar for all Socially Disadvantaged mother and infants over the first year of life. These findings raise critical questions about the specific social drivers of this effect for future studies, and policy decisions regarding resource allocation. It is a significant inequities issue, related to long-term health benefits for both mother an infant as recently identified by the American Academy of Pediatrics [27].

These SEM models elucidate: (1) how different indicators of adversity cohere to form factors of related indicators (i.e., Social Disadvantage and Psychosocial Stress) and (2) how disentangling components of adversity differentially contribute to gestational age and birthweight. The use of this modelling approach in a population sample enriched for adversity measured at each trimester of pregnancy revealed several additional findings. First, we did not see differential effects of exposures by trimester, in contrast to trimester specific effects of risk exposures described in the extant literature [28, 29]. However, in our sample, the indicators of Social Disadvantage and Psychosocial Stress were stable across trimesters, which may have precluded us from identifying trimester-specific effects. However, it is also possible that trimester effects will become evident in later infant outcomes. Second, it is notable that we did not find relationships of maternal psychosocial distress as indexed by depression, perceived distress, experience of discrimination, and life events once indicators of Social Disadvantage were included in the model for either gestational age or birthweight. These findings contrast with previous work reporting relationships of maternal depression and stress to neonatal outcomes [4]. However, many of these prior studies did not account for socioeconomic indicators aligned with distress, or use samples enriched for adversity.

### Strengths and limitations

Despite similar levels of adversity between our sample and several reported in the literature, our relatively comprehensive assessment of adversity and stress experiences modeled using SEM revealed that, at least in terms of gestational age and birthweight, the socio-economic factors played a more central role. However, as indicated in the model, adversity and psychosocial stress are closely related with adversity being a likely source of psychosocial stress. It is possible that stronger relationships to psychosocial stress will emerge when other childhood outcomes are examined, including brain development, behavior, emotional, and physical health. In addition, our sample utilized self-report measures of depression rather than diagnostic measures, and rates were somewhat lower than those in samples of clinically depressed pregnant women, which might have led to smaller than expected effects in this domain. Third, we were not able to investigate the role of race in this analysis due to the high co-linearity between race and SES in this sample. While race and class are highly linked in US samples, it is clear that race and class also intersect in important ways that relate to experiences of discrimination and social exclusion, factors which will be an important area of focus in this study sample as we follow offspring over time [30].

## Conclusions

These data highlight the central importance of maternal experiences of social disadvantage during pregnancy in predicting key early infant outcomes– gestational age and birthweight as well as breastfeeding practices. Given that both gestational age and birthweight and breastfeeding are infant outcomes known to predict numerous later health trajectories, these findings further validate the principle that the social determinants of health are initiated during pregnancy and are powerful in predicting health outcomes. Further, this model provides an organizational framework to inform how the effects of adversity/disadvantage might be accounted for in studies of prenatal programming. Studies from these data have utilizing factors derived from this model have found significant relationships of social disadvantage and psychosocial stress to neonatal brain outcomes at birth [31, 32]. Future studies will focus on behavioral and brain outcomes over the course of early childhood development. Current findings highlight the importance of these forms of adversity/disadvantage and their differential contributions to key neonatal birth outcomes. Findings suggest that SD in pregnancy should now be recognized as a key health prevention target in prenatal health care. Programs that target pregnant women facing high SD including income infusion and socio-emotional support are promising prevention targets and their effects on infant outcomes should now be tested.

## Summary

### What’s known on this subject

- The extant literature has demonstrated effects of adversity and separately of psychosocial stress in pregnancy on infant outcomes. However, how these risk factors interact and whether they impact fetal development when maternal health risks are accounted for remains unclear.

### What this study adds

- This study elucidates the relationship of social adversity and psychosocial stress on infant outcomes when other key associated risk factors are considered. Study findings highlight the need to address social adversity during pregnancy as a key infant health prevention target.

## Supplementary information

Supplemental Materials

## Data Availability

Data availability upon request.
