# Supplementary material for: Social disadvantage during pregnancy: effects on gestational age and birthweight
Source: J Perinatol. 2023 Mar 13;43(4):477–83. doi: 10.1038/s41372-023-01643-2 (PMC10079545; doi:10.1038/s41372-023-01643-2)
Supplement: Supplementary file 1 — Supplemental Materials [file 41372_2023_1643_MOESM1_ESM.docx]

**Supplemental Materials**

Supplemental materials include additional details about the indicators of maternal social disadvantage and psychosocial stress, additional details about the statistical methods and results for the structural equation models generating latent factors for maternal social disadvantage and psychosocial stress, tables that detail the results of the structural equation models for each outcome, and another set of analyses that accounts for substance use in the sample.

**Supplemental Methods**

*Maternal Social Disadvantage Indicators*: Income to Needs ratio (I/N) was measured at each trimester. The I/N ratio utilizes self-reported family income and household size compared to federal poverty thresholds, with a ratio of 1.0 defining the poverty line. A log_10_ transform was applied to the ratio to make it symmetric around 1.0 and to deal with skewed distribution of the actual ratio. Insurance status was collected at the time of enrollment through a medical record review and was verified in the third trimester or at delivery. Mother’s highest level of education was self-reported at the time of enrollment. Area Deprivation Index (ADI), a geotracking measure, was used to rank neighborhoods by socioeconomic disadvantage compared to the national average based on census block data, including factors for the domains of income, education, employment, and housing quality.^1^ Maternal nutrition over the past year was assessed in the third trimester or at birth using the Healthy Eating Index (HEI), a validated dietary assessment tool used to measure diet quality based on U.S Dietary Guidelines for Americans with scores ranging from 0 to 100 with the average American score 59.^2^ Dietary information for HEI calculation was obtained using the Diet History Questionnaire (DHQII).^3^

*Maternal Psychosocial Stress Indicators:* In each trimester, mothers completed the Edinburgh Postnatal Depression Scale (EPDS)^4^ and Perceived Stress scale (PSS)^5^. The Stress and Adversity Inventory (STRAIN)^6^, a comprehensive measure assessing participants’ lifetime exposure to stressful and traumatic experiences, was administered at the time of neonatal MRI scan (N=255) or at a follow-up exam (N=108); no differences in STRAIN scores based on time of administration were found. Experiences of discrimination based on race were assessed using the Everyday Discrimination Scale^7^ measured at neonatal MRI scan.

**Supplemental Statistical Methods and Results**

To adjust birthweight for gestational age, we fit a Loess regression model using the default parameters in Proc LOESS of SAS. The residuals from this regression were used as the dependent measure assessing the degree that the weight of the infant was heavier or lighter than average for gestational age in this sample. Because of observed heteroscedasticity, a log_10_ transformation of birthweight was used.

In fitting the models, we examined the residual correlations which remained unexplained by the pre-specified model and added covariances where necessary. In particular, we examined whether any of the variables associated with the latent factors were also related to gestational age or birthweight not accounted for by the latent factor, which would indicate a direct effect in addition to that contributed by the latent variable. The STDY standardization of the effects was used for all continuous variables. The variances of the Social Disadvantage and Psychosocial factors were set at 1.0.

We compared this two-factor model to a one-factor model where none of the fit indices were in an acceptable range (RMSEA=0.14, SRMR=0.15, CFI/TLI=0.512/0.408). A three-factor model had two fit indicators in an acceptable range (RMSEA=0.080, SMSR=0.080), but CFI/TLI was 0.844/0.808 and not in an acceptable range with loadings on the third factor all low. Importantly, there were low levels of correlations of the measured variables for one factor (e.g., Social Disadvantage) with the other factor (Table 2; Tables S2 and S3). Notably, for the two-factor models.

Note that the loadings in Table 2 are slightly different for indicators of the two latent constructs across models predicting gestational age and birthweight given that multiple regression SEM uses a non-linear optimization approach to fit each model.

**Supplemental Analyses Addressing Cannabis and Tobacco**

Cannabis and tobacco use can be more frequent among women who are experiencing social disadvantage and/or psychological distress and both cannabis and tobacco use have been associated with reduced birthweight. Thus, cannabis and/or tobacco use might be a confound in interpreting the relationships between social advantage and birthweight. Of the 399 women in this sample, 29 women reported using 1-5 cigarettes per day, and 21 reported 6 or more cigarettes per day, which was coded as 0/1/2 for analyses. Twenty women reported using cannabis at least one time weekly, but less than once a day, while 29 women reported using cannabis at least once a day (coded 0/1/2 for analyses). Of these women, 20 reported using both tobacco and cannabis. To determine whether social disadvantage continued to predict residual birthweight even accounting for cannabis and tobacco use, we created a revised SEM model. We constructed an SEM that included this association, and which allowed us to both examine cannabis and tobacco use as part of the pathway by which *Social Advantage* might be linked to birthweight (e.g., an indirect pathway), versus a direct pathway from *Social Advantage* independent of cannabis/tobacco use. As shown in Table S2, cannabis and tobacco use were significantly and strongly associated with the *Social Advantage* latent factor, and more modestly associated with the *Psychosocial Distress* latent factor. The total effect for the relationship of *Social Advantage* to residual birthweight (combination of both direct and indirect pathways) remained highly significantly. Critically, the direct relationship of *Social Advantage* (i.e., independent of tobacco and cannabis use) to residual birthweight remained significant, while there was no direct relationship of cannabis or tobacco use to birthweight independent of *Social Advantage*. These data indicate that while cannabis and tobacco use are greater among those women experiencing greater social disadvantage and psychosocial distress, social advantage still predicts birthweight even accounting for this substance use.

Table S1. Frequency timing and source of clinical and demographic variables

| **Variable** | **Frequency of measurement** | **Timing if singular** | **Composite value** | **Source** |
| --- | --- | --- | --- | --- |
| Income to Needs | Each trimester | NA | No | Self-reported |
| Insurance status | Singular | At enrollment | No | Medical Record |
| Maternal education | Singular | At enrollment | NA | Self-reported |
| ADI | Twice | At enrollment and third trimester | NA | Medical Record at enrollment and verified via self-report at third trimester |
| Healthy Eating Index | Singular | At third trimester or delivery | NA | DHQII self-reported |
| EPDS | Each trimester | NA | No | Self-reported |
| PSS | Each trimester | NA | No | Self-reported |
| STRAIN | Singular | At neonatal scan | No | Self-reported |
| Discrimination | Singular | At neonatal scan | No | Self-reported |
| Maternal age | Singular | At delivery | NA | Medical Record |
| Pre-pregnancy BMI | Singular | At enrollment | NA | Self-reported from Medical Record |
| MMR* | Preexisting conditions and over course of pregnancy | Enrollment to delivery | Yes 24 factors | Medical Record |
| Birthweight | Singular | At delivery | NA | Medical Record |
| Gestational age | Singular | At delivery | NA | Medical Record |

*Maternal co-morbidities included chronic congestive heart failure, congenital heart disease, pulmonary hypertension, chronic ischemic heart disease, cardiac valvular disease, pre-existing hypertension, gestational hypertension, chronic renal disease, preexisting diabetes mellitus, , asthma, severe pre-eclampsia, sickle cell disease, age >35, systemic lupus erythematousus, HIV virus, mild or unspecified pre-eclampsia, placental previa.

| **Table S2: Full Structural Equation Model Predicting Gestational Age** | | | | | | |
| --- | --- | --- | --- | --- | --- | --- |
| **Variable** | **Raw Estimate** | **STDY Estimate** | **Standard Error** | **Estimate/**  **Standard Error** | **p**  **(2-tailed)** | **Residual**  **Variance** |
| **Indicators of Social Disadvantage** | | | | | | |
| Income/Needs 1^st^ Trimester | -0.365 | -0.909 | 0.019 | -47.274 | 0.000 | 0.175 |
| Income/Needs 2^nd^ Trimester | -0.372 | -0.914 | 0.019 | -48.010 | 0.000 | 0.164 |
| Income/Needs 3^rd^ Trimester | -0.371 | -0.915 | 0.017 | -53.376 | 0.000 | 0.163 |
| Area Deprivation Index | 17.814 | 0.720 | 0.029 | 24.506 | 0.000 | 0.481 |
| Health Insurance | -2.833 | -0.842 | 0.029 | -28.962 | 0.000 |  |
| Education | -2.767 | -0.836 | 0.024 | -34.802 | 0.000 |  |
| HEI-2016 Total Score | -3.772 | -0.383 | 0.054 | -7.153 | 0.000 | 0.853 |
| **Indicators of Psychosocial Distress** | | | | | | |
| EPDS 1^st^ Trimester | 3.638 | 0.748 | 0.033 | 22.593 | 0.000 | 0.441 |
| EPDS 2^nd^ Trimester | 3.822 | 0.769 | 0.031 | 24.953 | 0.000 | 0.409 |
| EPDS 3^rd^ Trimester | 3.395 | 0.708 | 0.051 | 13.923 | 0.000 | 0.498 |
| PSS 1^st^ Trimester | 5.713 | 0.772 | 0.029 | 26.571 | 0.000 | 0.405 |
| PSS 2^nd^ Trimester | 6.317 | 0.811 | 0.030 | 26.857 | 0.000 | 0.343 |
| PSS 3^rd^ Trimester | 5.357 | 0.712 | 0.038 | 18.641 | 0.000 | 0.493 |
| STRAIN - CT | 2.611 | 0.432 | 0.051 | 8.499 | 0.000 | 0.813 |
| STRAIN – WT SEV | 9.325 | 0.471 | 0.051 | 9.208 | 0.000 | 0.778 |
| Discrimination Survey | 0.217 | 0.248 | 0.057 | 4341 | 0.000 | 0.938 |
| **Predictors of Gestational Age** | | | | | | 0.835 |
| Social Disadvantage | -0.429 | -0.216 | 0.072 | -2.996 | 0.003 |  |
| Psychosocial Distress | -0.070 | -0.035 | 0.057 | -0.622 | 0.534 |  |
| Maternal Medical Risk Score | -0.348 | -0.295 | 0.073 | -4.043 | 0.000 |  |
| Maternal Age at Delivery | -0.013 | -0.035 | 0.066 | -0.529 | 0.597 |  |
| Pre-Pregnancy BMI | -0.009 | -0.037 | 0.059 | -0.633 | 0.527 |  |
| Cervix Length | 0.037 | 0.141 | 0.071 | 1.978 | 0.048 |  |
| **Covariances** | | | | | | |
| **Computed from confirmatory factor analysis** | |  |  |  |  |  |
| Social Disadvantage w Psychosocial Distress | | 0.354 | 0.045 | 7.856 | 0.000 |  |
| **Inserted because of residual correlations** | |  |  |  |  |  |
| Income/Needs 1^st^ Trimester w 2^nd^ Trimester | | 0.727 | 0.063 | 11.527 | 0.000 |  |
| Income/Needs 1^st^ Trimester w 3^rd^ Trimester | | 0.668 | 0.068 | 9.855 | 0.000 |  |
| Income/Needs 2^nd^ Trimester w 3^rd^ Trimester | | 0.753 | 0.054 | 14.053 | 0.000 |  |
| STRAIN – STRAIN-CT w STRAIN-WTSEV | | 0.917 | 0.011 | 86.779 | 0.000 |  |
| **Covariances among predictors** | |  |  |  |  |  |
| Maternal Medical Risk w Social Disadvantage | | -0.008 | 0.050 | -0.167 | 0.867 |  |
| Maternal Medical Risk w Psychosocial Distress | | 0.011 | 0.052 | 0.211 | 0.833 |  |
| Maternal Medical Risk w Maternal Age at Delivery | | 0.278 | 0.047 | 5.860 | 0.000 |  |
| Maternal Medical Risk w Pre-Pregnancy BMI | | 0.237 | 0.059 | 4.024 | 0.000 |  |
| Maternal Medical Risk w Cervix Length | | 0.008 | 0.045 | 0.173 | 0.863 |  |
| Maternal Age at Delivery w Social Disadvantage | | -0.430 | 0.046 | -9.349 | 0.000 |  |
| Maternal Age at Delivery w Psychosocial Distress | | -0.195 | 0.054 | -3.647 | 0.000 |  |
| Maternal Age at Delivery w Pre-Pregnancy BMI | | 0.089 | 0.060 | 1.472 | 0.141 |  |
| Maternal Age at Delivery w Cervix Length | | 0.066 | 0.058 | 1.142 | 0.254 |  |
| Pre-Pregnancy BMI w Social Disadvantage | | 0.152 | 0.058 | 2.628 | 0.009 |  |
| Pre-Pregnancy BMI w Psychosocial Distress | | 0.081 | 0.062 | 1.302 | 0.193 |  |
| Pre-Pregnancy BMI w Cervix Length | | 0.037 | 0.082 | 0.454 | 0.650 |  |
| Cervix Length w Social Disadvantage | | 0.042 | 0.058 | 0.719 | 0.472 |  |
| Cervix Length w Psychosocial Distress | | -0.023 | 0.057 | -0.399 | 0.690 |  |

| **Table S3: Full Structural Equation Model Predicting Birthweight Adjusted for Gestational Age** | | | | | | |
| --- | --- | --- | --- | --- | --- | --- |
| **Variable** | **Raw Estimate** | **STDY Estimate** | **Standard Error** | **Estimate/**  **Standard Error** | **p**  **(2-tailed)** | **Residual**  **Variance** |
| **Indicators of Social Disadvantage** | | | | | | |
| Income/Needs 1^st^ Trimester | -0.363 | -0.903 | 0.019 | -47.856 | 0.000 | 0.184 |
| Income/Needs 2^nd^ Trimester | -0.369 | -0.909 | 0.019 | -48.297 | 0.000 | 0.174 |
| Income/Needs 3^rd^ Trimester | -0.368 | -0.909 | 0.017 | -53.274 | 0.000 | 0.174 |
| Area Deprivation Index | 17.847 | 0.722 | 0.029 | 24.545 | 0.000 | 0.479 |
| Health Insurance | -2.873 | -0.846 | 0.029 | -29.369 | 0.000 |  |
| Education | -2.828 | -0.842 | 0.023 | -36.015 | 0.000 |  |
| HEI-2016 Total Score | -3.756 | -0.381 | 0.054 | -7.125 | 0.000 | 0.854 |
| **Indicators of Psychosocial Distress** | | | | | | |
| EPDS 1^st^ Trimester | 3.637 | 0.747 | 0.033 | 22.618 | 0.000 | 0.441 |
| EPDS 2^nd^ Trimester | 3.824 | 0.769 | 0.031 | 25.064 | 0.000 | 0.408 |
| EPDS 3^rd^ Trimester | 3.395 | 0.708 | 0.051 | 13.930 | 0.000 | 0.498 |
| PSS 1^st^ Trimester | 5.710 | 0.771 | 0.029 | 26.572 | 0.000 | 0.405 |
| PSS 2^nd^ Trimester | 6.313 | 0.810 | 0.030 | 26.862 | 0.000 | 0.343 |
| PSS 3^rd^ Trimester | 5.353 | 0.711 | 0.038 | 18.613 | 0.000 | 0.494 |
| STRAIN - CT | 2.614 | 0.433 | 0.051 | 8.519 | 0.000 | 0.813 |
| STRAIN – WT SEV | 9.331 | 0.472 | 0.051 | 9.205 | 0.000 | 0.778 |
| Discrimination Survey | 0.218 | 0.249 | 0.057 | 4.344 | 0.000 | 0.938 |
| **Predictors of Birthweight** | | | | | | 0.940 |
| Social Disadvantage | -0.012 | -0.201 | 0.073 | -2.770 | 0.006 |  |
| Psychosocial Distress | -0.001 | -0.020 | 0.055 | -0.367 | 0.711 |  |
| Maternal Medical Risk Score | 0.000 | -0.013 | 0.053 | -0.241 | 0.810 |  |
| Maternal Age at Delivery | 0.000 | 0.022 | 0.060 | 0.364 | 0.716 |  |
| Pre-Pregnancy BMI | 0.001 | 0.143 | 0.061 | 2.352 | 0.019 |  |
| HEI-2016 Total Score | 0.000 | 0.005 | 0.058 | 0.087 | 0.931 |  |
| **Covariances** | | | | | | |
| **Computed from confirmatory factor analysis** | |  |  |  |  |  |
| Social Disadvantage w Psychosocial Distress | | 0.354 | 0.045 | 7.855 | 0.000 |  |
| **Inserted because of residual correlations** | |  |  |  |  |  |
| Income/Needs 1^st^ Trimester w 2^nd^ Trimester | | 0.742 | 0.057 | 13..115 | 0.000 |  |
| Income/Needs 1^st^ Trimester w 3^rd^ Trimester | | 0.687 | 0.060 | 11.403 | 0.000 |  |
| Income/Needs 2^nd^ Trimester w 3^rd^ Trimester | | 0.767 | 0.048 | 16.080 | 0.000 |  |
| STRAIN – STRAIN-CT w STRAIN-WTSEV | | 0.916 | 0.011 | 86.703 | 0.000 |  |
| **Covariances among predictors** | |  |  |  |  |  |
| Maternal Medical Risk w Social Disadvantage | | -0.009 | 0.050 | -0.182 | 0.856 |  |
| Maternal Medical Risk w Psychosocial Distress | | 0.011 | 0.052 | 0.210 | 0.834 |  |
| Maternal Age at Delivery w Social Disadvantage | | -0.434 | 0.046 | -9.464 | 0.000 |  |
| Maternal Age at Delivery w Psychosocial Distress | | -0.196 | 0.054 | -3.644 | 0.000 |  |
| Pre-Pregnancy BMI w Social Disadvantage | | 0.153 | 0.058 | 2.626 | 0.009 |  |
| Pre-Pregnancy BMI w Psychosocial Distress | | 0.085 | 0.062 | 1.381 | 0.167 |  |
| Pre-Pregnancy BMI w Maternal Medical Risk | | 0.236 | 0.059 | 4.016 | 0.000 |  |
| Pre-Pregnancy BMI w Maternal Age at Delivery | | 0.090 | 0.060 | 1.505 | 0.132 |  |
| Maternal Age at Delivery w Maternal Medical Risk | | 0.278 | 0.047 | 5.860 | 0.000 |  |

**Table S4: SEM Model Predicting Gestational Age Incorporating Cannabis and Tobacco Use**

| **Variable** | **Raw Estimate** | **STDY Estimate** | **Standard Error** | **Estimate/**  **Standard Error** | **p**  **(2-tailed)** | **Residual**  **Variance** |
| --- | --- | --- | --- | --- | --- | --- |
| **Indicators of Social Disadvantage** | | | | | | |
| Income/Needs 1^st^ Trimester | -0.364 | -0.906 | 0.019 | -48.814 | 0.000 | 0.179 |
| Income/Needs 2^nd^ Trimester | -0.371 | -0.914 | 0.018 | -49.760 | 0.000 | 0.165 |
| Income/Needs 3^rd^ Trimester | -0.370 | -0.914 | 0.016 | -55.439 | 0.000 | 0.165 |
| Area Deprivation Index | 17.735 | 0.717 | 0.029 | 24.611 | 0.000 | 0.486 |
| Health Insurance | -2.869 | -0.845 | 0.029 | -29.231 | 0.000 |  |
| Education | -2.793 | -0.839 | 0.024 | -35.464 | 0.000 |  |
| HEI-2016 Total Score | -3.804 | -0.386 | 0.053 | -7.226 | 0.000 | 0.851 |
| **Indicators of Psychosocial Distress** | | | | | | |
| EPDS 1^st^ Trimester | 3.646 | 0.749 | 0.033 | 22.941 | 0.000 | 0.439 |
| EPDS 2^nd^ Trimester | 3.824 | 0.769 | 0.031 | 25.072 | 0.000 | 0.408 |
| EPDS 3^rd^ Trimester | 3.384 | 0.706 | 0.051 | 13.843 | 0.000 | 0.502 |
| PSS 1^st^ Trimester | 5.726 | 0.773 | 0.029 | 26.560 | 0.000 | 0.402 |
| PSS 2^nd^ Trimester | 6.323 | 0.811 | 0.030 | 26.954 | 0.000 | 0.342 |
| PSS 3^rd^ Trimester | 5.344 | 0.709 | 0.039 | 18.371 | 0.000 | 0.497 |
| STRAIN - CT | 2.622 | 0.434 | 0.051 | 8.551 | 0.000 | 0.812 |
| STRAIN – WT SEV | 9.355 | 0.473 | 0.051 | 9.263 | 0.000 | 0.777 |
| Discrimination Survey | 0.217 | 0.248 | 0.057 | 4.324 | 0.000 | 0.938 |
| **Predictors of Gestational Age** | | | | | | 0.825 |
| Social Disadvantage | -0.516 | -0.260 | 0.077 | -3.379 | 0.001 |  |
| Psychosocial Distress | -0.100 | -0.051 | 0.055 | -0.911 | 0.362 |  |
| Maternal Medical Risk Score | -0.352 | -0.299 | 0.073 | -4.116 | 0.000 |  |
| Maternal Age at Delivery | -0.025 | -0.063 | 0.068 | -0.925 | 0.355 |  |
| Pre-Pregnancy BMI | -0.006 | -0.025 | 0.059 | -0.416 | 0.678 |  |
| Cervix Length | 0.038 | 0.146 | 0.067 | 2.180 | 0.029 |  |
| Cannabis | 0.064 | 0.018 | 0.056 | 0.320 | 0.749 |  |
| Tobacco | 0.411 | 0.104 | 0.042 | 2.445 | 0.014 |  |
| **Covariances** | | | | | | |
| **Computed from confirmatory factor analysis** | |  |  |  |  |  |
| Social Disadvantage w Psychosocial Distress | | 0.355 | 0.045 | 7.869 | 0.000 |  |
| **Inserted because of residual correlations** | |  |  |  |  |  |
| Income/Needs 1^st^ Trimester w 2^nd^ Trimester | | 0.732 | 0.060 | 12.278 | 0.000 |  |
| Income/Needs 1^st^ Trimester w 3^rd^ Trimester | | 0.674 | 0.063 | 10.635 | 0.000 |  |
| Income/Needs 2^nd^ Trimester w 3^rd^ Trimester | | 0.755 | 0.051 | 14.695 | 0.000 |  |
| STRAIN – STRAIN-CT w STRAIN-WTSEV | | 0.916 | 0.011 | 86.771 | 0.000 |  |
| **Covariances among predictors** | |  |  |  |  |  |
| Maternal Medical Risk w Social Disadvantage | | -0.009 | 0.050 | -0.173 | 0.863 |  |
| Maternal Medical Risk w Psychosocial Distress | | 0.010 | 0.052 | 0.197 | 0.844 |  |
| Maternal Medical Risk w Maternal Age at Delivery | | 0.278 | 0.047 | 5.860 | 0.000 |  |
| Maternal Medical Risk w Pre-Pregnancy BMI | | 0.236 | 0.059 | 4.025 | 0.000 |  |
| Maternal Medical Risk w Cannabis | | 0.066 | 0.055 | 1.204 | 0.228 |  |
| Maternal Medical Risk w Tobacco | | 0.066 | 0.054 | 1.226 | 0.220 |  |
| Maternal Medical Risk w Cervix Length | | 0.008 | 0.045 | 0.186 | 0.852 |  |
| Maternal Age at Delivery w Social Disadvantage | | -0.431 | 0.046 | -9.446 | 0.000 |  |
| Maternal Age at Delivery w Psychosocial Distress | | -0.196 | 0.054 | -3.648 | 0.000 |  |
| Maternal Age at Delivery w Pre-Pregnancy BMI | | 0.091 | 0.060 | 1.511 | 0.131 |  |
| Maternal Age at Delivery w Cannabis | | -0.082 | 0.048 | -1.716 | 0.086 |  |
| Maternal Age at Delivery w Tobacco | | 0.068 | 0.049 | 1.396 | 0.163 |  |
| Maternal Age at Delivery w Cervix Length | | 0.063 | 0.057 | 1.093 | 0.275 |  |
| Pre-Pregnancy BMI w Social Disadvantage | | 0.150 | 0.058 | 2.597 | 0.009 |  |
| Pre-Pregnancy BMI w Psychosocial Distress | | 0.079 | 0.062 | 1.268 | 0.205 |  |
| Pre-Pregnancy BMI w Cannabis | | -0.003 | 0.057 | -0.059 | 0.953 |  |
| Pre-Pregnancy BMI w Tobacco | | -0.012 | 0.057 | -0.212 | 0.832 |  |
| Pre-Pregnancy BMI w Cervix Length | | 0.027 | 0.083 | 0.331 | 0.741 |  |
| Cannabis w Social Disadvantage | | 0.279 | 0.026 | 10.669 | 0.000 |  |
| Cannabis w Psychosocial Distress | | 0.225 | 0.054 | 4.152 | 0.000 |  |
| Cannabis w Tobacco | | 0.319 | 0.073 | 4.364 | 0.000 |  |
| Cannabis w Cervix Length | | -0.108 | 0.061 | -1.785 | 0.074 |  |
| Tobacco w Social Disadvantage | | 0.289 | 0.031 | 9.467 | 0.000 |  |
| Tobacco w Psychosocial Distress | | 0.202 | 0.056 | 3.626 | 0.000 |  |
| Tobacco w Cervix Length | | 0.001 | 0.069 | 0.019 | 0.985 |  |
| Cervix Length w Social Disadvantage | | 0.045 | 0.058 | 0.780 | 0.435 |  |
| Cervix Length w Psychosocial Distress | | -0.023 | 0.057 | -0.401 | 0.689 |  |

**Table S5: SEM Model Predicting Birthweight Controlling for Gestational Age Incorporating Cannabis and Tobacco Use**

| **Variable** | **Raw Estimate** | **STDY Estimate** | **Standard Error** | **Estimate/**  **Standard Error** | **p**  **(2-tailed)** | **Residual**  **Variance** |
| --- | --- | --- | --- | --- | --- | --- |
| **Indicators of Social Disadvantage** | | | | | | |
| Income/Needs 1^st^ Trimester | -0.364 | -0.904 | 0.019 | -48.079 | 0.000 | 0.182 |
| Income/Needs 2^nd^ Trimester | -0.371 | -0.912 | 0.019 | -48.856 | 0.000 | 0.168 |
| Income/Needs 3^rd^ Trimester | -0.370 | -0.912 | 0.017 | -54.220 | 0.000 | 0.169 |
| Area Deprivation Index | 17.733 | 0.717 | 0.029 | 24.428 | 0.000 | 0.486 |
| Health Insurance | -2.903 | -0.848 | 0.029 | -29.691 | 0.000 |  |
| Education | -2.837 | -0.842 | 0.023 | -35.901 | 0.000 |  |
| HEI-2016 Total Score | -3.767 | -0.382 | 0.054 | -7.141 | 0.000 | 0.854 |
| **Indicators of Psychosocial Distress** | | | | | | |
| EPDS 1^st^ Trimester | 3.648 | 0.750 | 0.033 | 23.037 | 0.000 | 0.438 |
| EPDS 2^nd^ Trimester | 3.826 | 0.770 | 0.031 | 25.220 | 0.000 | 0.407 |
| EPDS 3^rd^ Trimester | 3.384 | 0.706 | 0.051 | 13.878 | 0.000 | 0.502 |
| PSS 1^st^ Trimester | 5.725 | 0.773 | 0.029 | 26.577 | 0.000 | 0.402 |
| PSS 2^nd^ Trimester | 6.319 | 0.810 | 0.030 | 26.977 | 0.000 | 0.343 |
| PSS 3^rd^ Trimester | 5.337 | 0.708 | 0.039 | 18.284 | 0.000 | 0.498 |
| STRAIN - CT | 2.632 | 0.436 | 0.051 | 8.572 | 0.000 | 0.810 |
| STRAIN – WT SEV | 9.380 | 0.474 | 0.051 | 9.263 | 0.000 | 0.775 |
| Discrimination Survey | 0.217 | 0.248 | 0.057 | 4.323 | 0.000 | 0.938 |
| **Predictors of Birthweight** | | | | | | 0.925 |
| Social Disadvantage | -0.008 | -0.138 | 0.070 | -1.984 | 0.047 |  |
| Psychosocial Distress | 0.001 | 0.009 | 0.055 | 0.162 | 0.871 |  |
| Maternal Medical Risk Score | 0.000 | -0.003 | 0.050 | -0.068 | 0.945 |  |
| Maternal Age at Delivery | 0.001 | 0.061 | 0.063 | 0.979 | 0.328 |  |
| Pre-Pregnancy BMI | 0.001 | 0.125 | 0.060 | 2.083 | 0.037 |  |
| Cannabis | -0.010 | -0.088 | 0.052 | -1.680 | 0.093 |  |
| Tobacco | -0.013 | -0.105 | 0.061 | -1.730 | 0.084 |  |
| **Covariances** | | | | | | |
| **Computed from confirmatory factor analysis** | |  |  |  |  |  |
| Social Disadvantage w Psychosocial Distress | | 0.355 | 0.045 | 7.868 | 0.000 |  |
| **Inserted because of residual correlations** | |  |  |  |  |  |
| Income/Needs 1^st^ Trimester w 2^nd^ Trimester | | 0.737 | 0.058 | 12.615 | 0.000 |  |
| Income/Needs 1^st^ Trimester w 3^rd^ Trimester | | 0.681 | 0.062 | 11.065 | 0.000 |  |
| Income/Needs 2^nd^ Trimester w 3^rd^ Trimester | | 0.759 | 0.050 | 15.208 | 0.000 |  |
| STRAIN – STRAIN-CT w STRAIN-WTSEV | | 0.916 | 0.011 | 86.657 | 0.000 |  |
| **Covariances among predictors** | |  |  |  |  |  |
| Maternal Medical Risk w Social Disadvantage | | -0.024 | 0.059 | -0.415 | 0.678 |  |
| Maternal Medical Risk w Psychosocial Distress | | 0.020 | 0.061 | 0.330 | 0.742 |  |
| Maternal Age at Delivery w Social Disadvantage | | -0.409 | 0.050 | -8.189 | 0.000 |  |
| Maternal Age at Delivery w Psychosocial Distress | | -0.045 | 0.056 | -0.806 | 0.420 |  |
| Pre-Pregnancy BMI w Social Disadvantage | | 0.131 | 0.068 | 1.920 | 0.055 |  |
| Pre-Pregnancy BMI w Psychosocial Distress | | 0.022 | 0.072 | 0.312 | 0.755 |  |
| Cannabis w Social Disadvantage | | 0.231 | 0.033 | 6.925 | 0.000 |  |
| Cannabis w Psychosocial Distress | | 0.147 | 0.060 | 2.451 | 0.014 |  |
| Tobacco w Social Disadvantage | | 0.241 | 0.037 | 6.484 | 0.000 |  |
| Tobacco w Psychosocial Distress | | 0.121 | 0.062 | 1.944 | 0.052 |  |

**Table S6: Logistic Regressions of NICU Stay and Breastfeeding Outcomes by Prenatal Social Disadvantage and Psychosocial Stress**

| **DV = NICU Stay (N=321)** | **Estimate** | **SE** | **χ^2^** | **p** |
| --- | --- | --- | --- | --- |
| Maternal age at birth | -0.0011 | 0.0414 | 0.00 | 0.9792 |
| Gestational age | -0.9575 | 0.1706 | 31.50 | <0.0001 |
| Child birthweight | 0.0007 | 0.0005 | 2.12 | 0.1456 |
| Maternal medical risk | 0.2025 | 0.1063 | 3.63 | 0.0568 |
| Pre-pregnancy BMI | -0.0124 | 0.0253 | 0.24 | 0.6246 |
| Social disadvantage | 0.1644 | 0.2669 | 0.38 | 0.5380 |
| Psychosocial stress | 0.1049 | 0.2085 | 0.25 | 0.6150 |
| **DV = Breastmilk ever in NICU (N=51)** | **Estimate** | **SE** | **χ^2^** | **p** |
| Maternal age at birth | 0.0045 | 0.0713 | 0.00 | 0.9495 |
| Gestational age | 0.0036 | 0.1997 | 0.00 | 0.9856 |
| Child birthweight | -0.0005 | 0.0007 | 0.50 | 0.4783 |
| Maternal medical risk | -0.1131 | 0.1330 | 0.72 | 0.3951 |
| Pre-pregnancy BMI | 0.0965 | 0.0679 | 2.02 | 0.1551 |
| Social disadvantage | -0.3506 | 0.5025 | 0.49 | 0.4854 |
| Psychosocial stress | -0.5380 | 0.4081 | 1.74 | 0.1874 |
| **DV = Breastmilk at NICU discharge (N=51)** | **Estimate** | **SE** | **χ^2^** | **p** |
| Maternal age at birth | -0.0355 | 0.0671 | 0.28 | 0.5969 |
| Gestational age | 0.2298 | 0.1948 | 1.39 | 0.2382 |
| Child birthweight | 0.0000 | 0.0007 | 0.00 | 0.9566 |
| Maternal medical risk | 0.1222 | 0.1216 | 1.01 | 0.3149 |
| Pre-pregnancy BMI | 0.0427 | 0.0451 | 0.90 | 0.3439 |
| Social disadvantage | -0.9397 | 0.4605 | 4.16 | 0.0413 |
| Psychosocial stress | -0.4404 | 0.4006 | 1.21 | 0.2716 |
| **DV = Breastmilk at 4 months (N=258)** | **Estimate** | **SE** | **χ^2^** | **p** |
| NICU stay | -0.1913 | 0.2575 | 0.55 | 0.4576 |
| Maternal age at birth | 0.0674 | 0.0341 | 3.91 | 0.0480 |
| Gestational age | 0.0453 | 0.1257 | 0.13 | 0.7183 |
| Child birthweight | 0.0006 | 0.0004 | 3.01 | 0.0825 |
| Maternal medical risk | -0.0491 | 0.1053 | 0.22 | 0.6412 |
| Pre-pregnancy BMI | -0.0366 | 0.0207 | 3.11 | 0.0777 |
| Social disadvantage | -0.8958 | 0.2010 | 19.86 | <0.0001 |
| Psychosocial stress | 0.1558 | 0.1686 | 0.85 | 0.3554 |
| **DV = Breastmilk at 1 year (N=252)** | **Estimate** | **SE** | **χ^2^** | **p** |
| NICU stay | 0.0844 | 0.2611 | 0.10 | 0.7464 |
| Maternal age at birth | 0.0261 | 0.0369 | 0.50 | 0.4806 |
| Gestational age | -0.0163 | 0.1142 | 0.02 | 0.8868 |
| Child birthweight | 0.0006 | 0.0003 | 3.30 | 0.0694 |
| Maternal medical risk | -0.2670 | 0.1221 | 4.79 | 0.0287 |
| Pre-pregnancy BMI | -0.0166 | 0.0194 | 0.73 | 0.3929 |
| Social disadvantage | -0.5910 | 0.1853 | 10.17 | 0.0014 |
| Psychosocial stress | -0.1636 | 0.1779 | 0.85 | 0.3579 |

**Supplemental References**

1. Kind AJ, Buckingham WR. Making neighborhood-disadvantage metrics accessible—the neighborhood atlas. *The New England journal of medicine.* 2018;378(26):2456.

2. Chen E, Miller GE, Kobor MS, Cole SW. Maternal warmth buffers the effects of low early-life socioeconomic status on pro-inflammatory signaling in adulthood. *Mol Psychiatry.* 2011;16(7):729-737.

3. Diet History Questionnaire, Version 2.0. In: National Institutes of Health, Epidemiology and Genomics Research Program, National Cancer Institute; 2010.

4. Cox JL, Holden JM, Sagovsky R. Detection of postnatal depression. Development of the 10-item Edinburgh Postnatal Depression Scale. *Br J Psychiatry.* 1987;150:782-786.

5. Cohen S, Kamarck T, Mermelstein R. Perceived stress scale. *Measuring stress: A guide for health and social scientists.* 1994;10:1-2.

6. Slavich GM, Shields GS. Assessing lifetime stress exposure using the Stress and Adversity Inventory for Adults (Adult STRAIN): An overview and initial validation. *Psychosomatic Medicine.* 2018;80(1):17.

7. Lewis TT, Yang FM, Jacobs EA, Fitchett G. Racial/ethnic differences in responses to the everyday discrimination scale: a differential item functioning analysis. *American journal of epidemiology.* 2012;175(5):391-401.
